# Investigation on left ventricular multi-directional deformation in patients of hypertension with different LVEF

**DOI:** 10.1186/s12947-017-0106-7

**Published:** 2017-06-12

**Authors:** Huimei Huang, Qinyun Ruan, Meiyan Lin, Lei Yan, Chunyan Huang, Liyun Fu

**Affiliations:** Department of Ultrasound, the First Affiliated Hospital of Fujian Medical University, Fuzhou, 350005 China

**Keywords:** Hypertension, Heart failure, Myocardial contraction, Left ventricular ejection fraction, Strain, Echocardiography

## Abstract

**Background:**

This study is aimed at investigating myocardial multi-directional systolic deformation in hypertensive with different left ventricular ejection fraction (LVEF), and exploring its contribution to LVEF.

**Methods:**

One hundred and twenty-three patients with primary hypertension (HT) were divided into group A (LVEF ≥ 55%), group B (45% ≤ LVEF < 50%, or 50% ≤ LVEF < 55% + LVEDVI ≥ 97 ml/m^2^), and group C (LVEF < 45%). Two-dimensional strain echocardiography (2DSE) including LV longitudinal strain (SL), radial strain (SR) and circumferential strain (SC) were measured.

**Results:**

SL decreased gradually from group A, B to C (all *p* < 0.05) while SR and SC were reduced only in group B and C (all *p* < 0.05). All strain measurements correlated to LVEF, with the strongest correlation in SC (*r* = −0.82, *p* < 0.01) and the second in SL (*r* = −0.76). The diastolic E/e increased from group A, B to C.

**Conclusions:**

Left ventricular multi-directional deformation correlated well to LVEF in hypertension and particularly SC, indicating that it was SC, not SL or SR, that makes the prominent contribution to left ventricular pump function.

## Background

Heart failure with preserved ejection fraction (HFpEF) and heart failure with reduced EF (HFrEF) may be different stages of the identical disease [1]. Studies in patients of HFpEF revealed a general trend of a decrease in left ventricular ejection fraction (LVEF) although it remains in “normal range”. In addition, there are abnormalities in other indices of left ventricular (LV) systolic function such as systolic mitral annulus velocity and displacement, LV myocardial strain and strain rate obtained by tissue doppler imaging (TDI) and 2-dimensional strain echocardiography (2DSE) [2–5]. Previous work including the one we conducted have shown a depressed LV longitudinal strain in the early stages of hypertensive LV remodeling (concentric remodeling and concentric hypertrophy) [6, 7]. Impaired LV longitudinal strain occurs in some patients with hypertension and normal LVEF in the absence of heart failure [8]. However, with the progression of LV dysfunction, the characteristic of myocardial deformations in all directions is unknown. This study aimed to investigate myocardial multi-directional strain in hypertensive patients with normal, borderline and reduced left ventricular ejection fraction (LVEF), thus to explore the contribution of myocardial multi-directional deformation to LVEF.

## Method

### Study population

According to 2007 Guidelines for the management of arterial hypertension [9], hypertension was defined as systolic blood pressure ≥ 140 mmHg or diastolic blood pressure ≥ 90 mmHg. Inclusion criteria: One hundred and twenty-three patients (from October 2014 to October 2015) who had hypertension as defined above were enrolled in. Normal controls (NC) contains 40 age and sex matched volunteers who were free of cardiovascular or systemic diseases. Patients were excluded for the following reasons: poor image quality, atrial fibrillation, known coronary artery disease, regional wall motion abnormality in left ventricle, idiopathic cardiomyopathy, congenital heart disease, chest distress, exertional angina pectoris. According to LVEF, the subjects were divided into two groups: hypertension with normal LVEF (HT-NEF, LVEF ≥ 50%) and hypertension with reduced LVEF (HT-REF, LVEF < 50%). In order to investigate a “borderline” state, all the subjects were further divided into three sub-groups: group A, normal LVEF and LV end diastolic volume index (LVEF ≥ 55%, LVEDVI < 97 ml/m^2^); group B, “borderline” LVEF and enlarged LV end diastolic volume index (45% ≤ LVEF < 50%, or 50% ≤ LVEF < 55% + LVEDVI ≥ 97 ml/m^2^) and group C, reduced LVEF (LVEF < 45%).

### Conventional Echocardiographic study

Echocardiography was performed in participants using GE VIVID E9 or GE VIVID E7 ultrasound scanner (GE Vingmed Ultrasound, Horten, Norway) with GE VIVID E9 or GE VIVID E7 probe (1.7-4.0 MHz). Conventional scans were acquired in standard left ventricular long axis, short axis and apical views. For parasternal short-axis views, three levels of LV were acquired, which were basal level, papillary muscle level and apical level. All echocardiographic measurements were averaged on three heart beats. Left ventricular ejection fraction (LVEF), left ventricular end-diastolic volume (LVEDV) and left atrial volume (LAV) were obtained by area-length method in standard apical views (biplane). Left ventricular internal dimension at end diastole and end systole (LVIDd, LVIDs), posterior wall thickness (LVPWT), septal thickness (IVST) and left atrial diameter (LAD) were acquired in parasternal long axis views. Left ventricular mass (LVM) was calculated based on the recently published guidelines [10]. LVEDV, LAV and LVM were indexed to body surface area (BSA). E/A ratio were measured from mitral inflow (measured at the tips of the mitral valve), peak early (E) and late (A) filling velocities. Tissue Doppler was applied at end-expiration in the pulsed-wave doppler mode at the level of the mitral annulus from an apical four-chamber view. Lateral and septal mitral annulus early diastolic velocities (e’) were recorded and averaged to derive E/e’ ratio. LV hypertrophy was defined as an LVMI ≥ 125 g/m^2^ for men and LVMI ≥ 110 g/m^2^ for women [9]. LV enlargement [10] was defined as an LVEDVI ≥ 97 ml/m^2^.

### LV systolic strain measurements by 2DSE

Myocardial strain measurements were performed using 2DSE [11–13]. The analysis was performed offline using commercial software (EchoPAC Software, version 113, General Electric Company, Horten, Norway). To optimize speckle tracking, 2D gray-scale harmonic images were obtained at a frame rate of >50 frames/s. In above software, myocardial deformation measurements were performed using tissue speckle tracking and the displacement of speckles of myocardium in each spot was analyzed and tracked frame by frame. After manual tracing of the endocardial border of the end-systolic frame and selecting the appropriate region of interest, i.e. the width of the region of interest was adjusted to fit the wall thickness as required, the software automatically determined six segments in each view. Each segmental strain curve was obtained by automatic frame-by-frame tracking of the acoustic markers in the myocardial tissue. The tracking quality was scored as either acceptable or unacceptable. For each subject, longitudinal strain values for all six LV myocardial segments in each of the apical four-chamber views were measured and averaged to derive the global LV longitudinal strain (SL). Circumferential and radial strain values were obtained in all 18 segments at the level of the three short-axis views. The average of peak systolic circumferential or radial strain values from the three short-axis views was calculated to derive global LV circumferential strain and radial strain (SC, SR). All the measurements were performed in triplicate and averaged from three regular heart beats. Segments with obvious bias tracking were excluded from the analysis.

### Interobserver and Intraobserver variability

Ten patients were randomly-selected to assess interobserver and intraobserver variability in strain measurements. The interobserver variability was calculated as the SD of the difference between the measurements of two independent observers who were blinded to all other patient’s data and expressed as a percent of the average value. The intraobserver variability was calculated as the SD of the difference between the first and second measurements by the same observer at 1-week interval and expressed as a percent of the average value.

### Statistical analysis

Continuous data were presented as mean ± standard deviation (SD) or as percentages where appropriate. Comparisons among multiple groups was performed with one-way ANOVA if the data were normally distributed; otherwise, one-way ANOVA on ranks if the data distribution was not normal. Categorical variables were analyzed using χ^2^ test, and Fisher’s exact tests were used when appropriate. Pearson’s correlation analysis was used to study the relation between two continuous variables. SPSS version 15 (IBM Corporation, Armonk, NY) was deployed to perform the majority of the statistical operations, where a *p* value less than 0.05 was considered statistically significant.

## Results

### Clinical characteristics and Echocardiographic measurements in HT

There was no difference in terms of age or sex among all patients groups and normal controls (*p* > 0.05), as shown in Table 1.

**Table 1:** Clinical characteristics and conventional echocardiographic measurements in hypertensive

	NC	HT-NEF (LVEF ≥ 50%)	HT-REF (LVEF < 50%)
	*n* = 40	*n* = 81	*n* = 42
Age(y)	53.75 ± 11.72	57.25 ± 11.70	57.55 ± 14.69
Gender(M/F)	26/14	60/21	36/6
LBBB/RBBB(n)	0	3	6
PAB(n)	0	3	1
PVB(n)	0	2	2
Abnormal blood lipid or DM(n)	0	2	10^△▲^
Renal dysfunction(n)	0	4	3
HR(beats/min)	67.60 ± 10.17	69.35 ± 11.90	78.33 ± 15.47^△▲^
SBP(mmHg)	122.48 ± 13.66	151.30 ± 19.91^△^	145.37 ± 25.80^△^
DBP(mmHg)	78.14 ± 10.44	88.48 ± 13.64^△^	89.11 ± 14.56^△^
LVIDd(cm)	4.70 ± 0.28	5.20 ± 0.56^△^	5.98 ± 0.86^△▲^
IVST (cm)	0.87 ± 0.12	1.19 ± 0.30^△^	1.22 ± 0.31^△^
LVPWT(cm)	0.76 ± 0.09	1.02 ± 0.21^△^	1.08 ± 0.24^△^
RWT	0.35 ± 0.04	0.43 ± 0.10^△^	0.40 ± 0.12
LVMI(g/m^2^)	76.41 ± 10.74	131.79 ± 44.35^△^	175.77 ± 49.84^△▲^
LAD(cm)	3.17 ± 0.59	3.97 ± 0.57^△^	4.39 ± 0.65^△▲^
LAVI(ml/ m^2^)	23.05 ± 8.69	31.72 ± 9.67^△^	40.01 ± 17.20^△▲^
FS(%)	34.29 ± 3.47	34.59 ± 4.25	18.43 ± 5.10^△▲^
LVEDVI(ml/ m^2^)	62.45 ± 8.60	76.14 ± 16.24^△^	109.06 ± 40.15^△▲^
LVEF(%)	62.56 ± 4.61	61.37 ± 5.20	37.30 ± 8.37^△▲^
PVA(m/s)	0.62 ± 0.20	0.77 ± 0.21^△^	0.70 ± 0.26
PVE(m/s)	0.64 ± 0.21	0.65 ± 0.21	0.73 ± 0.29
E/A	1.08 ± 0.39	0.96 ± 0.78	1.28 ± 0.90^▲^
E/e	7.53 ± 2.39	11.12 ± 4.16^△^	17.07 ± 11.20^△▲^
s(m/s)	0.07 ± 0.01	0.07 ± 0.03	0.05 ± 0.02^△▲^
a(m/s)	0.09 ± 0.02	0.09 ± 0.05	0.07 ± 0.08^▲^
e(m/s)	0.09 ± 0.03	0.07 ± 0.06^△^	0.05 ± 0.02^△▲^

Data are expressed as mean ± standard deviation (SD) or as number (ratio);

*NC* normal controls, *LVEF* left ventricular ejection fraction, *HT-NEF* hypertension with normal, *LVEF; HT-REF* hypertension with reduced LVEF, *HT* hypertension, *LBBB* left bundle branch block, *RBBB* right bundle branch block, *PAB* occasional premature atrial beats, *PVB* occasional premature ventricular beats, *DM* diabetes mellitus, *HR* heart rate, *SBP* systolic blood pressure, *DBP* diastolic blood pressure, *LVIDd* Left ventricular internal dimension in diastole, *IVST* Interventricular septal thickness, *LVPWT* Left ventricular posterior wall thickness, *RWT* Relative wall thickness, *LVM* Left ventricular mass, *LVMI* Left ventricular mass index, *LAD* Left atrial diameter, *LAVI* LA volume index, *FS* Fractional shortening, *LVEDVI* Left ventricular end-diastolic volume index, *LVEF* Left ventricular ejection fraction;

^△^:*p* < 0.05 vs NC group; ^▲^:*p* < 0.05 vs group HT-NEF

Besides reduced LVEF, patients in group HT-REF had lower annular systolic velocity and higher LAVI, LVIDd, LVMI, LVEDVI (*p* < 0.05); early diastolic e’ velocity decreased and E/e’ ratio increased progressively from NC to group HT-NEF and HT-REF (for each *p* < 0.05 in Table 1); there were no differences between group HT-NEF and NC in all strain measurements except that SL decreased significantly, while all the strain measurements were significantly lower in group HT-REF compared to group HT-NEF(*p* < 0.05, Table 2).

**Table 2:** LV strain measurements in group HT-NEF and HT-REF

	NC	HT-NEF (LVEF ≥ 50%)	HT-REF (LVEF < 50%)
	*n* = 40	*n* = 81	*n* = 42
SL(%)	−20.16 ± 2.75	−18.27 ± 3.74^△^	−11.34 ± 3.85^△▲^
SC-mv(%)	−18.31 ± 2.91	−17.54 ± 3.60	−10.54 ± 3.59^△▲^
SC-pm(%)	−18.01 ± 2.79	−18.14 ± 3.02	−11.09 ± 4.12^△▲^
SC-ap(%)	−21.37 ± 14.71	−23.01 ± 6.96	−15.01 ± 6.65^△▲^
SR-mv(%)	43.99 ± 19.47	40.69 ± 14.48	20.42 ± 8.61^△▲^
SR-pm(%)	42.75 ± 18.53	46.90 ± 16.95	22.83 ± 12.66^△▲^
SR-ap(%)	22.84 ± 17.10	19.78 ± 17.22	11.94 ± 8.17^△▲^

Data expressed as mean ± standard deviation (SD); *LV* left ventricular, *NC* normal controls, *LVEF* left ventricular ejection fraction, *HT-NEF* hypertension with normal LVEF, *HT-REF* hypertension with reduced LVEF, *SL* longitudinal strain, *SC* circumferential strain, *SR* radial strain; −mv, −pm, −ap indicates basal level, papillary and apical level;

^△^ *p* < 0.05 vs NC; ^▲^ *p* < 0.05 vs group HT-NEF

### Echocardiographic measurements in three HT sub-groups

**Fig. 1:**
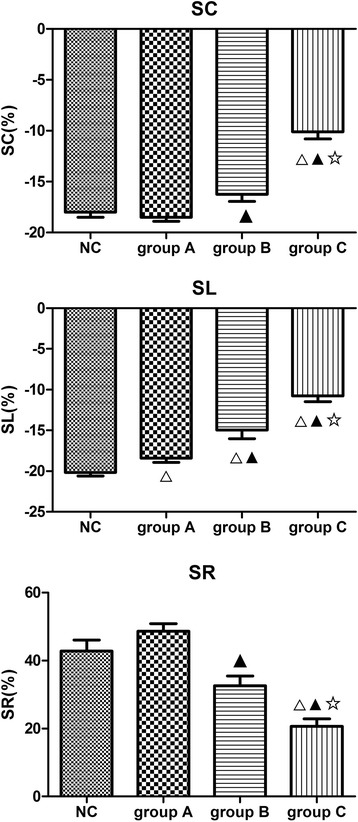
Comparison of multi-directional strains (papillary level) among NC and three subgroups divided according to LVEF. Note that all the strain measurements showed a downward trend from group A to B to C, where SL was impaired in group A and was further impaired in group B and C. SC and SR were impaired in group B and C (all *p* < 0.05).△ *p* < 0.05 vs NC group; ▲ *p* < 0.05 vs group A; ☆ *p* < 0.05 vs group B

Dilated LV and LA and increased LVMI appeared in all sub-groups with a progression from group A, B to C along with a diastolic in e’ velocity and an increase in E/e’ ratio. All the strain measurements except for apical SR showed a downward trend from group A, B to C, where SL was impaired in group A and was further impaired in group B and C. SC and SR were impaired in group B and C (all *p* < 0.05, Table 3, Fig. 1).

**Table 3:** Clinical characteristics and echocardiographic measurements of three sub-groups

	group A	group B	group C
	*n* = 71	*n* = 13	*n* = 34
Age(y)	57.99 ± 11.94	56.31 ± 14.07	57.62 ± 14.42
Gender(M/F)	53/18	11/2	28/6
HR(beats/min)	69.54 ± 11.97	66.92 ± 9.67	80.50 ± 15.81^△▲☆^
LVIDd(cm)	5.17 ± 0.56^△^	5.61 ± 0.47^△▲^	6.10 ± 0.88^△▲☆^
RWT	0.44 ± 0.10^△^	0.41 ± 0.11	0.38 ± 0.12^▲^
LVEDVI(ml/ m^2^)	74.61 ± 15.56^△^	91.14 ± 19.56^△▲^	114.97 ± 41.34^△▲☆^
LVMI(g/ m^2^)	132.09 ± 44.70^△^	151.45 ± 42.37^△^	181.44 ± 51.20^△▲☆^
LAD(cm)	3.99 ± 0.56^△^	4.11 ± 0.66^△^	4.45 ± 0.64^△▲☆^
LAVI(ml/m^2^)	31.33 ± 9.19^△^	31.91 ± 9.76^△^	42.69 ± 17.71^△▲☆^
LVEF(%)	62.65 ± 4.14	49.45 ± 2.51^△▲^	34.79 ± 7.24^△▲☆^
s(m/s)	0.07 ± 0.03	0.06 ± 0.02	0.05 ± 0.02^△▲^
E/e	11.13 ± 4.17^△^	11.19 ± 3.79	18.76 ± 11.78^△▲☆^
e(m/s)	0.07 ± 0.06	0.06 ± 0.03^△^	0.05 ± 0.02^△▲^

Data are expressed as mean ± standard deviation (SD) or as number (ratio); *HR* heart rate, *LVIDd* Left ventricular internal dimension in diastole, *RWT* Relative wall thickness, *LVEDVI* Left ventricular end-diastolic volume index, *LVMI* Left ventricular mass index, *LAD* Left atrial diameter, *LAVI* LA volume index, *LVEF* Left ventricular ejection fraction; the data of normal controls (NC) see Table one; group A, normal LVEF and LV end diastolic volume index, group B, “borderline” LVEF and enlarged LV end diastolic volume index, group C, reduced LVEF (LVEF < 45%). ^△^ *p* < 0.05 vs NC; ^▲^ *p* < 0.05 vs group A; ^☆^ *p* < 0.05 group B

### Correlation analysis of strains with LVEF

**Fig. 2:**
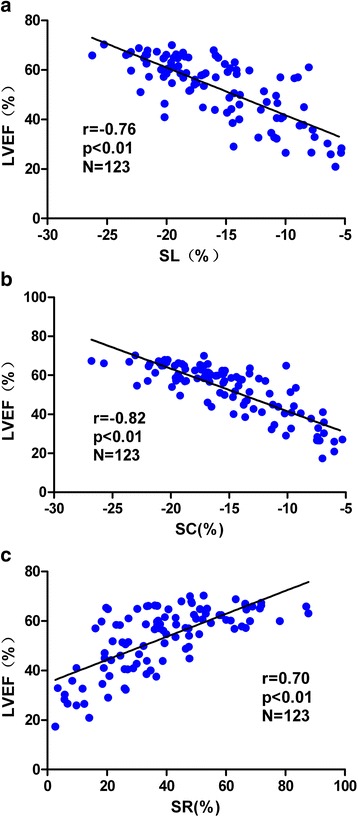
Correlation analysis of the strains to LVEF (**a**, **b** and **c** showing SL, SC, and SR were highly related with LVEF, with the strongest correlation in SC). (Note that because SL and SC was in negative value, while LVEF was positive, as a result, r-value was negative)

We analyzed the correlations of systolic multi-directional strain with LVEF and found a good connection in all strains, where the correlation coefficients in SC, SL and SR were −0.82, −0.76 and 0.70 respectively in Fig. 2.

## Discussion

In the present study, we compared the difference of LV multi-directional strain among patients in three different levels of LVEF, and investigated the correlation and contribution of strain in each direction to global pump function, providing a continuous trend of LV performance in the progression of cardiac dysfunction in hypertension. The major findings were, (1) SL decreased early in the stage of normal LVEF, while SC and SR began to decrease in patients with “borderline” LVEF (45%-55%) and deteriorated with LVEF reduced further. (2) Systolic strains in all directions highly correlated with LVEF, with the strongest correlation in SC (*r* = −0.82), indicating that it was SC, not SL or SR making the prominent contribution to left ventricular pump function.

### Left ventricular remodeling and function in hypertension

LVMI, LVEDVI and LAVI increased from group A, B to C. With the decrease of LVEF, annular e’ decreased together with s’, and E/e’ ratio increased. These results indicate that as the LV remodeling takes place, systolic performance is impaired together with diastolic dysfunction, which is in accordance with previous findings. In the progression from HFpEF to HFrEF, systolic dysfunction appeared while diastolic function deteriorated [3, 14]. HFpEF and HFrEF have similar pathophysiologic characteristics [14]. Biopsy of human myocardial tissue also showed HFpEF and HFrEF were injured to different degrees [15, 16], indicating that LV systolic and diastolic dysfunction are a continuous process, or two different stages in the progression of cardiac dysfunction [3].

### Left ventricular myocardial multi-directional deformation in hypertension

#### Longitudinal strain (SL)

Similar to several former studies, we found SL decreased in the early stage of hypertensive heart failure. Kosmala et al. found SL firstly reduced in NYHA I-II, and became lower as the cardiac function deteriorated [17]. Reduced SC, SR appeared only in NYHA III-IV. Another study reported impaired SL and SR in HFpEF [12].

#### Radial strain (SR)

We found SR was preserved or even increased slightly in early stage of hypertensive heart disease with normal LVEF and decreased in later stages (borderline and reduced LVEF). In the early stage of remodeling (concentric remodeling), or before left ventricular function is significantly impaired, SR is preserved or increased in compensation for the decrease in SL [6, 17]. As observed in the present study, the increased SR may be considered as a compensation for the impaired SL in early stage of hypertension [6]. In addition, the radius of curvature of the circumferentially oriented myocardial fibers responsible for LV radial deformation is smaller than that of longitudinal ones, which might entail lower stress and consequently delayed functional impairment caused by pressure overload [17]. SR derived from speckle tracking from endocardial to epicardial layer was not purely originated from the contraction of midwall fiber but the whole heart muscle layers, and was decreased when all heart muscle layers was injured [17–19].

#### Circumferential strain (SC)

The present results of SC were in accord with previous studies [6, 12, 13]: SC was preserved or slightly increased as a compensation to maintain a normal LVEF, the reduction of which may lead to a decreased LVEF. Investigation by Wang et al. had revealed that there was no difference between HFpEF and normal control in SC, while distinctly lowered SC only occurred in HFrEF [12]. Kouzu et al. found there was an increasing trend of SC in concentric remodeling, but a reduction in SC in patients with concentric hypertrophy and eccentric hypertrophy [6]. As mentioned above, mid-wall myocardial fibers were not affected in early stage and thus SC was preserved.

A recent study demonstrated that heart failure with normal LVEF had a poorer SC than normal subjects [8], where “normal LVEF” was defined as >45%, and the percentage of patients with abnormal SC was only 22% in patients of LVEF > 55%, lower than that with “borderline” LVEF (as described in our present study). In that study, patients in normal LVEF group mostly had a medical history of coronary disease or myocardial infarction which may indirectly lead to a decreased SC. In our present study, subjects with coronary artery disease or myocardial infarction were excluded, and we divided all the patients into 3 subgroups according to different LVEF, and we assessed LVEF using area-length method for better analysis of the correlation between LV multi-directional strain and global LV function.

### The pathophysiological mechanisms

SL was impaired first in the early stage, and deteriorated together with decreased SR and SC with the progression of hypertensive remodeling. The pathological basis of these observations likely lies in hypertension-related fibrosis and myocyte hypertrophy progressing from subendocardial layer to the epicardium [20], and the subendocardial fibers primarily affect longitudinal strain [21]. Importantly, biopsy of human myocardium shows interstitial subendocardial fibrosis in patients with HFpEF [15, 16]. With progression of disease to the mid and outer myocardial layers, circumferential, and radial strain also decline as was seen in patients with depressed LVEF.

### Correlations of strains to LVEF

The present study shows a significant correlation between longitudinal, circumferential, radial strain and LVEF. Impaired SL did not appear to impact LVEF because of the compensation of myocardial fibers in the mid and subepicardial layers. In the later stages of hypertension and with LV remodeling, other components of LV strain were also impaired [22]. In our study, SC related to LVEF best among all strains. Previous studies have also shown close correlation of SC or the time to peak of SC to LVEF [23, 24]. But some reports showed better correlation of SR with LVEF in patients of dilated cardiomyopathy in adolescents [25]. When LVEF is calculated from linear formula, where the only parameter used is from LV short axis dimension, SR was more strongly correlated with LVEF. In our study, LVEF was evaluated by area-length method, where LV stroke volume originated from the whole LV endocardial displacements caused by longitudinal, radial and circumferential systolic deformation, thus all directions of strain measurements had significant correlation with LVEF, with SC contributing more to LVEF. Geometrically, systolic SC comes from the contraction of circumferential fibers, leading to both systolic wall thickening (systolic SR) and decrease in LV diameter and volume. The preserved SC and LV twist may contribute to the normal LVEF in patients with HFpEF, and an increasing SC may be a compensatory mechanism to maintain a normal LVEF in the early stage [11, 12], and accordingly, the decrease of multi-directional deformation especially SC and the deterioration of diastolic function may lead to reduced LVEF and HFrEF.

## Limitations

We used 2DSE to reconstruct a 3D LV multi-directional strain, where images were obtained from different heart beats, and some detailed real myocardial dynamic information might be lost through heart motion in the chest in a cardiac cycle. Hence, the results might be less accurate than that of using a real time three dimensional strain. Since some LV short-axis views of apical level were challenging, we did not investigate LV twist, which would also contribute to preserved LVEF. We could not acquire detailed information of patients’ managements and medication of hypertension, for most of them did not have a regular management or they were not able to provide reliable records.

## Conclusions

Impaired left ventricular longitudinal strain and diastolic dysfunction happened early in patients with normal LVEF in hypertension, while circumferential and radial strains were preserved or increased slightly as a compensation for the decrease in local systolic function, and when all directions of strains were impaired, LVEF decreased together with a deteriorating LV diastolic function. Left ventricular multi-directional deformation correlated well to LVEF in hypertension, particularly SC, indicating that it was SC, not SL or SR making the prominent contribution to left ventricular pump function.

## References

[1] Paulus WJ, Tschope C, Sanderson JE (2007). How to diagnose diastolic heart failure: a consensus statement on the diagnosis of heart failure with normal left ventricular ejection fraction by the heart failure and echocardiography associations of the European Society of Cardiology. Eur Heart J.

[2] Yip G, Wang M, Zhang Y, Fung JW, Ho PY, Sanderson JE (2002). Left ventricular long axis function in diastolic heart failure is reduced in both diastole and systole: time for a redefinition?. Heart.

[3] Yu CM, Lin H, Yang H, Kong SL, Zhang Q, Lee S (2002). Progression of systolic abnormalities in patients with “isolated” diastolic heart failure. Circulation.

[4] Rosen BD, Edvardsen T, Lai S (2005). Left ventricular concentric remodeling is associated with decreased global and regional systolic function: the multi-ethnic study of atherosclerosis. Circulation.

[5] Carluccio E, Biagioli P, Alunni G (2011). Advantages of deformation indices over systolic velocities in assessment of longitudinal systolic function in patients with heart failure and normal ejection fraction. Eur J Heart Fail.

[6] Kouzu H, Yuda S, Muranaka A (2011). Left ventricular hypertrophy causes different changes in longitudinal, radial, and circumferential mechanics in patients with hypertension: a two-dimensional speckle tracking study. J Am Soc Echocardiogr.

[7] Lin MY, Ruan QY, Gai YY, Zeng KQ, Xie LD (2012). Investigation on myocardial systolic multi-dimensional deformation in hypertension patients with left ventricular remodeling by two-dimensional strain echocardiography. Chinese J Ultrasound Med.

[8] Kraigher-Krainer E, Shah AM, Gupta DK (2014). Impaired systolic function by strain imaging in heart failure with preserved ejection fraction. J Am Coll Cardiol.

[9] Mancia G, Backer GD, Dominiczak A (2007). 2007 guidelines for the management of arterial hypertension:the task force for the management of arterial hypertension of the European society of hypertension (ESH) and of the European society of cardiology (ESC). Eur Heart J.

[10] Lang RM, Badano LP, Mor-Avi V (2015). Recommendations for cardiac chamber quantification by echocardiography in adults: an update from the american society of echocardiography and the European association of cardiovascular imaging. J Am Soc Echocardiogr.

[11] Phan TT, Shivu GN, Shivu GH (2009). Left ventricular torsion and strain patterns in heart failure with normal ejection fraction are similar to age-related changes. Eur J Echocardiogr.

[12] Wang J, Khoury DS, Yue Y, Torre-Amione G, Nagueh SF (2008). Preserved left ventricular twist and circumferential deformation, but depressed longitudinal and radial deformation in patients with diastolic heart failure. Eur Heart J.

[13] Mizuguchi Y, Oishi Y, Miyoshi H, Iuchi A, Nagase N, Oki T (2008). The functional role of longitudinal, circumferential, and radial myocardial deformation for regulating the early impairment of left ventricular contraction and relaxation in patients with cardiovascular risk factors:a study with two-dimensional strain imaging. J Am Soc Echocardiogr.

[14] Kitzman DW, Little WC, Brubaker PH (2002). Pathophysiological characterization of isolated diastolic heart failure in comparison to systolic heart failure. JAMA.

[15] Heerebeek L, Borbely A, Niessen HW (2006). Myocardial structure and function differ in systolic and diastolic heart failure. Circulation.

[16] Borbely A, Velden J, Papp Z (2005). Cardiomyocyte stiffness in diastolic heart failure. Circulation.

[17] Kosmala W, Plaksej R, Strotmann JM (2008). Progression of left ventricular functional abnormalities in hypertensive patients with heart failure: an ultrasonic two-dimensional speckle tracking study. J Am Soc Echocardiogr.

[18] Rademakers FE, Rogers WJ, Guier WH (1994). Relation of regional cross-fiber shortening to wall thickening in the intact heart: three dimensional strain analysis by NMR tagging. Circulation.

[19] Waldman LK, Nosan D, Villarreal F, Covell JW (1988). Relation between transmural deformation and local myofiber direction in canine left ventricular. Circ Res.

[20] Ishizu T, Seo Y, Kameda Y (2014). Left ventricular strain and transmural distribution of structural remodeling in hypertensive heart disease. Hypertension.

[21] Cho GY, Marwick TH, Kim HS (2009). Global 2-dimensional strain as a new prognosticator in patients with heart failure. J Am Coll Cardiol.

[22] Lewis RP, Sandler H (1971). Relationship between changes in left ventricular dimensions and the ejection fraction in man. Circulation.

[23] Carasso S, Cohen O, Mutlak D (2011). Relation of myocardial mechanics in severe aortic stenosis to left ventricular ejection fraction and response to aortic valve replacement. Am J Cardiol.

[24] Ortega M, Triedman JK, Geva T, Harrild DM (2011). Relation of left ventricular dyssynchrony measured by cardiac magnetic resonance tissue tracking in repaired tetralogy of fallot to ventricular tachycardia and death. Am J Cardiol.

[25] Friedberg MK, Slorach C (2008). Relation between left ventricular regional radial function and radial wall motion abnormalities using two-dimensional speckle tracking in children with idiopathic dilated cardiomyopathy. Am J Cardiol.

